# GPR30 activation decreases anxiety in the open field test but not in the elevated plus maze test in female mice

**DOI:** 10.1002/brb3.197

**Published:** 2013-11-27

**Authors:** Divya Anchan, Sara Clark, Kevin Pollard, Nandini Vasudevan

**Affiliations:** 1 Neuroscience Program, Tulane UniversityNew Orleans, 70118, Louisiana; 2 Cell and Molecular Biology Department, Tulane UniversityNew Orleans, 70118, Louisiana

**Keywords:** Elevated plus maze, ERK, G-1, open field, rapid estrogen signaling

## Abstract

The GPR30 is a novel estrogen receptor (ER) that is a candidate membrane ER based on its binding to 17*β* estradiol and its rapid signaling properties such as activation of the extracellular-regulated kinase (ERK) pathway. Its distribution in the mouse limbic system predicts a role for this receptor in the estrogenic modulation of anxiety behaviors in the mouse. A previous study showed that chronic administration of a selective agonist to the GPR30 receptor, G-1, in the female rat can improve spatial memory, suggesting that GPR30 plays a role in hippocampal-dependent cognition. In this study, we investigated the effect of a similar chronic administration of G-1 on behaviors that denote anxiety in adult ovariectomized female mice, using the elevated plus maze (EPM) and the open field test as well as the activation of the ERK pathway in the hippocampus. Although estradiol benzoate had no effect on behaviors in the EPM or the open field, G-1 had an anxiolytic effect solely in the open field that was independent of ERK signaling in either the ventral or dorsal hippocampus. Such an anxiolytic effect may underlie the ability of G-1 to increase spatial memory, by acting on the hippocampus.

## Introduction

Anxiety disorders encompass a wide range of disorders, including panic disorders, obsessive-compulsive disorders, posttraumatic stress disorders, and generalized anxiety disorders (GAD) and have a 16% prevalence rate worldwide (Somers et al. 2006). As the incidence of anxiety disorders is twofold greater in females than males (Somers et al. 2006) and arises during puberty (Hayward and Sanborn 2002) (Angold and Worthman 1993), gonadal hormones, particularly estrogens (Sachar et al. 1976; Hamilton et al. 1984; Poromaa and Segebladh 2012), are thought to modulate mood. In female rodents, a number of studies have examined the effects of hormones using the open field test (OFT), the elevated plus maze (EPM), and the light–dark transition (LDT) test, which are paradigms of unconditioned conflict anxiety that model GAD (Uys et al. 2003). Proestrous rats (Frye et al. 2000) and mice (Walf et al. 2009) were less anxious on the EPM than diestrous animals suggesting that higher levels of ovarian hormones exert anxiolytic effects (Marcondes et al. 2001; Byrnes and Bridges 2006). However, this effect appears to depend on estradiol dose, timing (Slater and Blizard 1976), and even context. Although estradiol in OVX rats was an anxiolytic in a nonsocial EPM task, it exerted anxiogenic effects in a social interaction test with a same-gender partner, suggesting that estradiol may be an anxiolytic or an anxiogenic, depending on context (Koss et al. 2004). Proestrous levels of estrogen generated by a 25 *μ*g estradiol benzoate (EB) implant in OVX female mice also had anxiogenic effects in all three tests, that is, the EPM, LDT test, and the OFT (Morgan and Pfaff 2002). As these paradigms use chronic administration of estrogens, the classical estrogen receptor (ER) isoforms, ER*α* and ER*β*, are thought to slowly regulate anxiety via transcription (Nilsson et al. 2001). However, administration of 10 *μ*g/kg 17*β* estradiol exerted anxiolytic effects in the elevated T-maze within 30 min in OVX rats (Kalandakanond-Thongsong et al. 2012), whereas administration of 25 *μ*g/kg 17*β* estradiol to female mice was an anxiogenic in the EPM and open field (Kastenberger et al. 2012) tasks within 2 h of a single injection. These studies implicate a rapid, possibly nongenomic, mode of signaling by 17*β* estradiol that contributes to state anxiety. One candidate for nongenomic signaling by 17*β* estradiol is the GPR30, a former orphan G-protein coupled receptor that binds 17*β* estradiol with a *K*_d_ value of 6 nmol/L (Thomas et al. 2005). The expression of GPR30 in the hippocampus and the central amygdala (Hazell et al. 2009) suggests that this receptor contributes to some of 17*β* estradiol's effect on the limbic system. In OVX rats, chronic administration of the specific GPR30 agonist, G-1 at 5 *μ*g/day improved memory on a delayed matching to place (DMP) task that requires hippocampally encoded spatial memory (Hammond et al. 2009). In OVX acutely stressed mice, GPR30 expression increased in the basolateral amygdala and G-1 regulated the NMDA receptor system to increase inhibitory synaptic transmission (Tian et al. 2013), thus decreasing anxiety. Contrary to this anxiolytic effect of GPR30 activation, Kastenberger et al. (2012) showed that 1 mg/kg body weight of G-1 given 2 h before testing to OVX female mice increased anxiety in the EPM and OFT, but not in the LDT. Hence, similar to the studies that show both anxiolytic and anxiogenic effects of 17*β* estradiol, GPR30 activation by the use of a selective agonist also leads to differing effects on state anxiety that are dependent on dose and timing.

As recent studies suggest that enhanced performance on spatial tasks is correlated with lower anxiety (Kheirbek et al. 2013; Olsen et al. 2013), we hypothesized that the enhancement seen in the DMP task in OVX rats with chronic administration of G-1 (Hammond et al. 2009) could be due to an anxiolytic effect of GPR30 activation. Hence, adult ovariectomized mice chronically administered, via silastic implants, EB, G-1, or vehicle were tested on the EPM task and the open field test. Our second hypothesis was that the anxiolytic effect exerted by G-1 would correlate with increased extracellular-regulated kinase (ERK) activation as well as the subsequent phosphorylation of an ERK target – the serine 118 of the ER*α* itself (Kato et al. 1995) – in the hippocampus. This is because GPR30 activation increased ERK activation in a breast cancer (MCF-7) cell line (Filardo et al. 2000) and ERK signaling elevates mood (Einat et al. 2003; Qi et al. 2009) and cognition (Fernandez et al. 2008); this would then provide a molecular mechanism to explore in future work. We show here that chronic administration of G-1, but not EB, decreases anxiety in the OFT but not in the EPM, independent of the regulation of ERK and the S118 site on the ER*α* in either the dorsal or ventral hippocampus.

## Material and Methods

### Animals

Adult, wild-type C57/Bl6 female mice (14–18 weeks of age) were obtained from Charles River (Wilmington, MA). Mice were individually housed under a 12:12 light–dark cycle, and food and water were provided ad libitum. Cages were changed weekly and no more than 48 h before any test. All mice were ovariectomized under isoflurane anesthesia and received an injection of Buprenex (Reckitt Benckiser Pharmaceuticals, Inc., Richmond, VA) for postoperative analgesia. All mice were allowed to rest for 10 days following surgery to allow for recovery from surgery and reduction in circulating hormone levels. The weight of each mouse was tracked after each behavioral test and before sacrifice. All living conditions and tests were in accordance with the NIH Guide for the Care and Use of Laboratory Animals and approved by the Tulane University Institutional Animal Care and Use Committee.

### Hormone regimen

Ten days after OVX, mice were surgically implanted with subcutaneous silastic capsules (1.57 mm ID × 2.41 mm OD × 17 mm L; Dow Corning Corporation, Midland, MI) containing 20 *μ*L of sesame oil alone or 2 *μ*g of EB (Sigma-Aldrich Company, St. Louis, MO) or 10 *μ*g of G-1 (Tocris, Bristol, U.K.) (*n* = 12/treatment group). These numbers/treatment groups have been used in previous studies (Kastenberger et al. 2012). Silastic capsule preparation and implantation are performed as described in Moffatt et al. (1998) and Ogawa et al. (2003). Mice were allowed to recover for an additional 10 days before behavioral testing to achieve constant steady-state level of drug diffusion among treatment groups (Morgan and Pfaff 2002; Ogawa et al. 2003). All behavioral testing, once initiated, was performed within 20 days of implantation.

### Behavioral testing

All tests were conducted during the dark cycle, beginning 90 min after lights were turned off and after an acclimation period of at least 2 h to the testing room. A 3-day window was maintained between the EPM test and the OFT in order to avoid intertest effects. This timeline and method of testing follows previously published studies (Tomihara et al. 2009). The testing room was dimly lit by a red lamp with luminosity between 5 and 20 lux.

#### Elevated plus maze

The EPM apparatus consisted of four arms (31.25 cm L × 5 cm W × 14.5 cm H; Harvard Apparatus, Holliston, MA) at 90^o^ angles to each other with all arm platforms elevated 37.5 cm from the floor. At the start of a trial, the mouse was placed in the center with its nose directed toward the same closed arm and allowed to explore the maze freely for 5 min. The total time spent, total distance covered, and distance in and entries into each arm and the center were digitally recorded by the 2100 Plus Tracking System (HVS Image Limited, Mountain View, CA). Additional parameters determined in data analysis were latency to the open arm, average speed, as well as percentage of test time and distance spent in the open and closed arms.

#### Open field

The OFT was utilized to examine locomotor activity as well as anxious behavior. A 16-beam animal activity monitor was used to divide the Plexiglass arena (40 cm L × 40 cm W × 30 cm H) into center and periphery. Fusion software (AccuScan Instruments Incorporated, Columbus, OH) analyzed various parameters based on recorded activity, including total distance, entries, rest time, movement time, and latency to center and periphery. The movement time is the time spent by the animal moving, rather than freezing, in either the center or periphery. At the beginning of each test, every animal was introduced to the same corner (left back corner) of the arena and was allowed to explore the arena freely for 5 min. Animals were tested twice on consecutive days on the OFT to examine habituation.

### Sample collection and western blotting

Two days after the last test, animals were deeply anesthetized with isoflurane inhalation and rapidly decapitated. The uteri from the animals were removed and collected in preweighed tubes containing distilled water; wet weight and images of the whole dissected uteri were recorded. Dorsal and ventral hippocampus were dissected rapidly and homogenized in RIPA lysis buffer (Boston Bioproducts, Ashland, MA) containing both protease and phosphatase inhibitors (Sigma Aldrich, St. Louis, MO). Protein concentrations were determined by the Lowry assay (Bio-Rad, Hercules, CA). Total protein of 20 *μ*g was separated with sodiumdodecyl sulfate polyacrylamide gel electrophoresis (SDS-PAGE) using either 10% (for ERK and GAPDH) or 7.5% (for ER*α* and *α*-tubulin) acrylamide gels and then transferred to polyvinylidene difluoride membranes (Millipore, Billerica, MA). Membranes were blocked for 1 h at room temperature with 5% bovine serum albumin (BSA) in Tris-buffered saline with 0.1% Tween 20 (TTBS) followed by incubation with mild agitation with the following primary antibodies diluted in blocking buffer: antiphospho-p44/42 mitogen-activated protein kinase Thr202/Tyr204 (1:2500; #4377, Cell Signaling Technology, MA) for 1 h at room temperature, antiphospho-S118 ER*α* (1:5000; sc-12915-R, Santa Cruz Biotechnology, CA) overnight at 4 C, anti-ER*α* (H-184) (1:1000; sc-7207, Santa Cruz Biotechnology, CA) overnight at 4 C, and anti-*α* tubulin (1:10,000; 1878-1, Epitomics, CA) for 1 h at room temperature. Following primary incubation, blots were washed with TTBS and incubated with anti-rabbit or anti-mouse horseradish peroxidase (HRP)-conjugated secondary antibody (1:20,000; Cell Signaling Technology, MA) in 5% BSA/TTBS. Blots were washed with TTBS and incubated with SuperSignal West Femto Chemiluminescent Substrate (Thermo Scientific Inc, IL) and chemiluminescence recorded with the ChemiDoc XRS Imaging System (Bio-Rad Inc, CA). The phospho-ERK antibodies was stripped with Tris-HCl with 2% SDS and 0.114M 2-mercaptoethanol preheated to 55 C for 45 min. Stripped blots were again blocked in 5% BSA/TTBS and reprobed with anti-ERK-1 C-16 (1:2500; sc-93, Santa Cruz Biotechnology, CA) for 1 h at room temperature with mild agitation. Quantification of western blots was performed using the Quantity One 1-D Image Analysis Software (Bio-Rad). Integrated band intensity was calculated for each phosphorylated and total protein band in each lane and normalized to the GAPDH (for ERK kinase) or *α*-tubulin (for S118 phosphorylated ER*α* and total ER*α*) band within the lane for loading control. Experimental values were normalized to the vehicle-treated samples within the same blot for cross-blot comparison.

### Statistical analysis

GraphPad Prism 5.04 (GraphPad Software Incorporated, La Jolla, CA) was used to conduct all statistical analyses and for graphs. For the EPM, all behavioral parameters were analyzed for group differences using one-way analysis of variance (ANOVA) followed by the Bonferroni's post hoc test. For the open field test, a repeated measures 2-way ANOVA was used to compare between treatment group and day; Bonferroni's post hoc test shows differences between groups. Animals that were found to be outliers (defined as 2 SD from the mean) on multiple parameters were removed from analysis of all data due to the possibility that the implant may be the source of the variation; hence, animal numbers are not equal across treatment groups. In addition, Bartlett's test for equal variances was utilized to test for homogeneity for both behavioral tests. For western blots, group differences in average vehicle-normalized band intensity values were tested with one-way ANOVA followed by Tukey's post hoc test. Data are presented as mean ± SEM and a *P* < 0.05 was considered significant in all tests.

## Results

### Body weight and uterine weight

**Figure 1:**
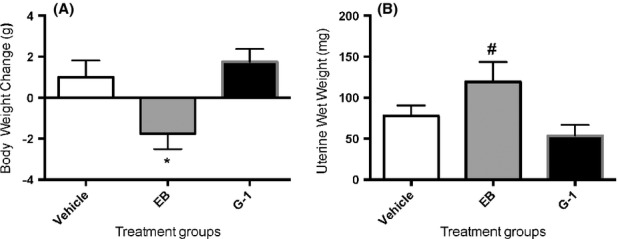
Estradiol benzoate (EB), but not G-1, decreases body weight but increases uterine weight. Animals were ovariectomized and implanted with silastic capsules that administered vehicle (sesame oil), 2 *μ*g EB, or 10 *μ*g G-1 per mouse. Animals were sacrificed after the end of behavioral testing and body and uteri weighed. Data represent mean ± SEM. *P < 0.05 cf vehicle. ^#^*P* < 0.05 cf G-1-treated group.

Animals were implanted with silastic capsules for 25 days and the difference in body weight between implantation and sacrifice was measured. EB-treated females were significantly lighter than vehicle-treated females (Fig. 1A); G-1-treated females were not significantly different from either vehicle-or EB-treated groups. G-1-treated animals also did not show any difference in uterine wet weight compared to control vehicle-treated animals. EB-treated animals showed a significant increase in uterine wet weight compared to G-1-treated animals (Fig. 1B).

### Behaviors that denote anxiety

**Figure 2:**
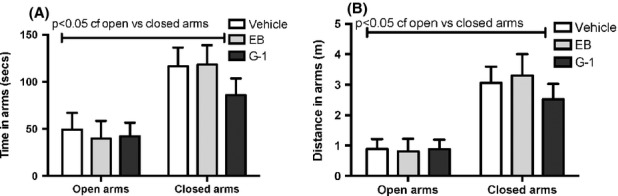
Ovariectomized females treated chronically with either 17*β*E_2_ or G-1 were tested in the elevated plus maze. No significant effect of treatment was found in either the time spent (Fig. 2A) or distance traveled (Fig. 2B) in either the open or closed arms. There was a significant increase in the time spent (sec) and the amount of distance (m) traveled in the closed arms compared to open arms across treatment groups. Data represent mean ± SEM.

**Figure 3:**
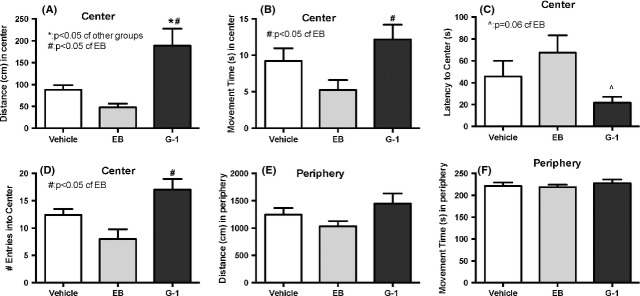
Ovariectomized females treated chronically with either 17*β*E_2_ or G-1 were tested in the open field arena. Mice treated chronically with G-1, but not EB, displayed a reduction in anxiety-like behaviors, traveling larger distances (cm) in the center of the arena (Fig. 3A) compared to vehicle and EB-treated mice (*P < 0.05), and more time (sec) moving in the center (Fig. 3B) compared to EB-treated mice (^#^*P* < 0.05). They also had an almost significant (*P* = 0.06) lower latency to the center (Fig. 3C) of the open field and a greater number of entries (Fig. 3D) in the open field than the EB-treated mice. No effect of treatment was revealed on the distance or time spent moving in the periphery. Data represent mean ± SEM.

In the EPM, there was no significant effect of treatment on the percentage of time or distance spent in closed or open arms (Fig. 2A and B). Across treatment groups, animals spent significantly more time and distance in closed arms compared to open arms (Fig. 2A and B). In the OFT, animals treated with G-1 moved a greater distance in the center of the open field compared to those treated with vehicle or EB (Fig. 3A). G-1-treated mice also spent more time moving in the center (Fig. 3B), had a lower latency to the center (Fig. 3C), and made more entries into the center (Fig. 3D) than the EB-treated group. However, there were no differences in distance and movement time in the periphery of the OFT by treatment group (Fig. 3E and F). On the second day of OFT testing, mice in all treatment groups traveled significantly less and spent less time in the center of the open field compared to the first day of testing (Fig. S1).

### Protein analysis

**Figure 4:**
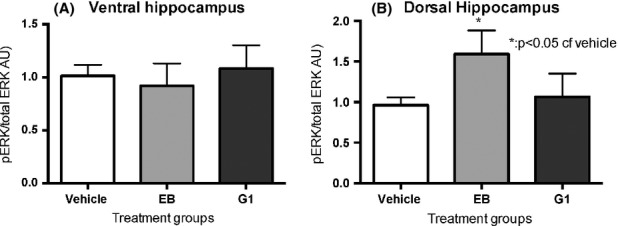
Chronic G-1 treatment does not regulate ERK activation. Animals administered EB or G-1 chronically in silastic implants were sacrificed, and western blot analysis on lysate from ventral hippocampus (A) and dorsal hippocampus (B) was performed. pERK is not regulated by hormone treatment in either the ventral or dorsal hippocampus by G-1 (A and B), but pERK is upregulated by EB in the dorsal (B) but not in the ventral hippocampus when compared to the vehicle-treated group. Data represent mean ± SEM.

**Figure 5:**
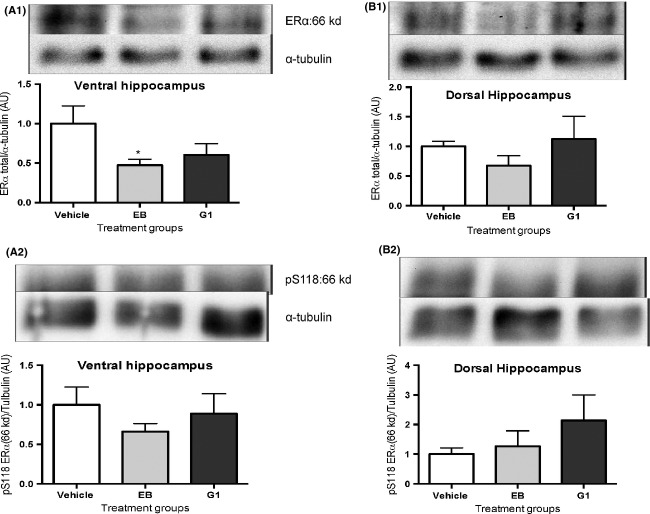
Although neither chronic EB nor G-1 treatment changed the phosphorylation at S118 in either the ventral hippocampus (A2) or dorsal hippocampus (B2), EB treatment decreased the ER*α* concentration in the ventral hippocampus (A1) but not in the dorsal hippocampus (B1). Animals were sacrificed and Western blotting analysis was performed on the lysate obtained from dorsal and ventral hippocampus. *P < 0.05 cf vehicle treatment. Data represent mean ± SEM.

We analyzed the activation of ERK1/2 by western blotting using specific antibodies to the ERK 202/204 site; phosphorylation of this site is required for activation. We also investigated phosphorylation at the serine 118 of the ER*α* using specific antibodies. Although there was no difference among treatment groups in the ventral hippocampus (Fig. 4A) in pERK levels, there was an increase in pERK in the dorsal hippocampus (Fig. 4B) in the EB-treated but not in the G-1-treated group, compared to the vehicle-treated group. In the ventral hippocampus but not in the dorsal hippocampus, there was a small decrease in total ER*α* in the EB-treated group compared to vehicle treatment (Fig. 5A); however, there were no differences among treatment groups in the phosphorylation of the ERK-dependent site at serine 118 (Fig. 5B) of the ER*α* in either the ventral or dorsal hippocampus.

## Discussion

### Doses of estrogen and G-1

Doses of estradiol as well as routes of administration in female rodents vary widely (Lund et al. 2005), depending on the study's endpoints. As we hypothesized that the chronic G-1 effect on cognition (Hammond et al. 2009) is linked to a possible anxiolytic effect of G-1, we chose an EB dose and a route – that is, silastic capsules to obtain steady-state levels – that could also lead to anxiolytic behavior, as a comparison. Our dose of 2 *μ*g EB/mouse translates into roughly 70 *μ*g/kg body weight, given a 30 g mouse at the time of testing. As this dose promoted lordosis in female mice when administered acutely 44 h before testing (White et al. 2007), we know that this dose acts on the brain. Also, OVX female rats administered EB doses ranging from 1 *μ*g/kg to 100 *μ*g/kg body weight chronically by daily injection for 4 weeks did not show graded anxiolytic responses in an elevated T-maze test that mirrored generalized anxiety disorder (Kalandakanond-Thongsong et al. 2012), suggesting that parameters that denote anxiety may not be sensitive to dose. However, a critical reason to choose lower rather than higher doses is that very low doses ∼0.1–0.2 *μ*g/mouse per day administered chronically had anxiolytic effects, whereas higher doses exerted anxiogenic effects (Tomihara et al. 2009). Our G-1 dose of 10 *μ*g/mouse translates to about 330 *μ*g/kg body weight, given a 30 g mouse; this is around five times more than the dose of EB/mouse. Although our chronic administration route and dose is not directly comparable to acute administration and dose of G-1 used in female mice (Kastenberger et al. 2012), a similar ratio of G-1:estradiol was used by Kastenberger et al. when acutely administering G-1 (at 1 mg/kg body weight) and 17*β*-estradiol (0.25 mg/kg body weight) to female OVX mice (Kastenberger et al. 2012). The GPR30 agonist, G-1, has been administered by s.c. injection to study acute effects (Kastenberger et al. 2012), and by osmotic pumps (Hammond et al. 2009) to study chronic effects. To the best of our knowledge, this is the first report where G-1 was delivered via implantation of a silastic capsule and had an effect on the central nervous system. As expected, EB decreased body weight (Windahl et al. 2009) and increased uterine wet weight (Gao et al. 2011); the lack of effect of G-1 in the uterus has been noted previously (Gao et al. 2011).

### EPM versus OFT

Both EPM and OFT are widely used as tasks that measure unconditioned avoidance of fearful situations (Donner and Lowry 2013) and are thought to model generalized anxiety disorder or GAD (Uys et al. 2003). As the cost of testing different groups of mice on each of the tests would be prohibitive, the mice were tested on the EPM first as it is the most sensitive test of anxiety (Ramos 2008). No treatment showed any differences when compared to vehicle in the EPM. However, surprisingly, although OFT conducted under red light is not deemed very fearful (DeFries et al. 1966), G-1 treatment produced an anxiolytic effect in this test, as can be seen by the greater distance and time spent in the center area of the novel arena. The lack of effect of G-1 in the EPM versus an anxiolytic effect in the OFT may be because of several reasons. C57BL/6J females are typically more anxious than males in the OFT (An et al. 2011), but less anxious than males in the EPM (Voikar et al. 2001), reflecting greater emotionality in the OFT rather than the EPM, suggesting that this strain responds to a greater extent in the OFT rather than the EPM. The effect of previous testing can also not be ruled out as individual animals could have different intrinsic anxiety at the time of the EPM versus the OFT (Ramos 2008). A third reason could be that the EPM and OFT may measure different aspects of anxiety. Although a pharmacological approach using benzodiazepine anxiolytics shows that both the EPM (Pellow et al. 1985) and OFT (Prut and Belzung 2003) are responsive to these drugs that regulate the GABAergic system, parameters that measure anxiety loaded onto different factors when rats (Ramos et al. 1998) and mice (Trullas and Skolnick 1993) were tested sequentially on the EPM and the OFT. Indeed, Ramos et al. (2008) found that even when the three tests were physically integrated into a single apparatus, the percentage of shared variance between paired variables such as the distance in the center in the OFT and the time in the open arms in the EPM was only 1.7%. Hence, these tests may measure different aspects of emotionality in mice. Finally, it is also possible that G-1 effect on anxiety is due to its effect on peripheral systems such as the cardiovascular system (Deschamps and Murphy 2009), which may influence state anxiety. The lack of EB regulation of anxiety in either the EPM or OFT is unclear, although it is possible that the EB effect in female mice may be more apparent under more stressful conditions such as white light, rather than the red light used in this study. Indeed, isolation stress resulted in an anxiolytic effect in the LDT but not in the EPM in female mice (Guo et al. 2004). In addition, the lack of an effect of EB on the OFT could be due to EB activation of multiple receptors that activate opposing signaling pathways while G-1 selectively activates solely GPR30 to give an anxiolytic phenotype.

### Signaling via ERK and ER

As ERK is involved in mood regulation (Einat et al. 2003) (Qi et al. 2009) and GPR30 activation can increase ERK signaling (Filardo et al. 2000), we hypothesized that regulation of anxiety may correlate with ERK activation in the ventral hippocampus as the ventral hippocampus is typically associated with anxiety (Alves et al. 2004). However, neither ERK nor a downstream substrate of ERK – the serine residue at position 118 of the ER*α* – is regulated by this dose of G-1 in either the dorsal or ventral hippocampus. This could be because the dose dependence of behavior versus that for signaling pathways could be different. For example, the total ER*α* concentration in the ventral hippocampus is decreased while the ERK activation in the dorsal hippocampus increased by EB treatment despite EB's lack of significant effect on any of the parameters in the EPM or the OFT. The ERK activation in the dorsal hippocampus by EB has been demonstrated to be required for learning in a novel object recognition task (Fernandez et al. 2008); however, the changes are more likely due to the hormone treatment as behaviors that denote anxiety in the EB-treated group did not change. However, both the increase in ERK and the decrease in ER*α* demonstrate that the lack of behaviors that denote increased or decreased anxiety in the EB-treated animals is not due to lack of EB entering the brain. It is also possible that other pathways activated by GPR30 such as protein kinase A (Thomas et al. 2005) or other nuclei such as the basolateral amygdala are important in anxiety regulation; these will be investigated in future studies.

### Summary

The anxiolytic effect seen with chronic GPR30 activation in the OFT could be beneficial for the improvement in cognition seen by other investigators (Hammond et al. 2009; Hammond and Gibbs 2011). The difference obtained with the tests underscores the importance of using more than one test in the investigation of state anxiety and suggests that GPR30-driven effects might be better revealed in females under less stressful conditions. Hence, selective activation of this receptor in the central nervous system is important for mood regulation.

## Supporting Information

Figure S1 Chronic EB or G-1 treatment does not affect habituation to the open field over consecutive days. Animals administered EB or G-1 chronically in silastic implants were tested on two consecutive days to investigate habituation to the open field arena. Both movement time (A) and distance (cm) decreased during Day 2 compared to Day 1, irrespective of hormone treatment (^*^P < 0.05 cf days). Data represent mean ± SEM.
