# Supplementary figures and images for: GPR30 activation decreases anxiety in the open field test but not in the elevated plus maze test in female mice
Source: Brain Behav. 2013 Nov 27;4(1):51–9. doi: 10.1002/brb3.197 (PMC3937706; doi:10.1002/brb3.197)

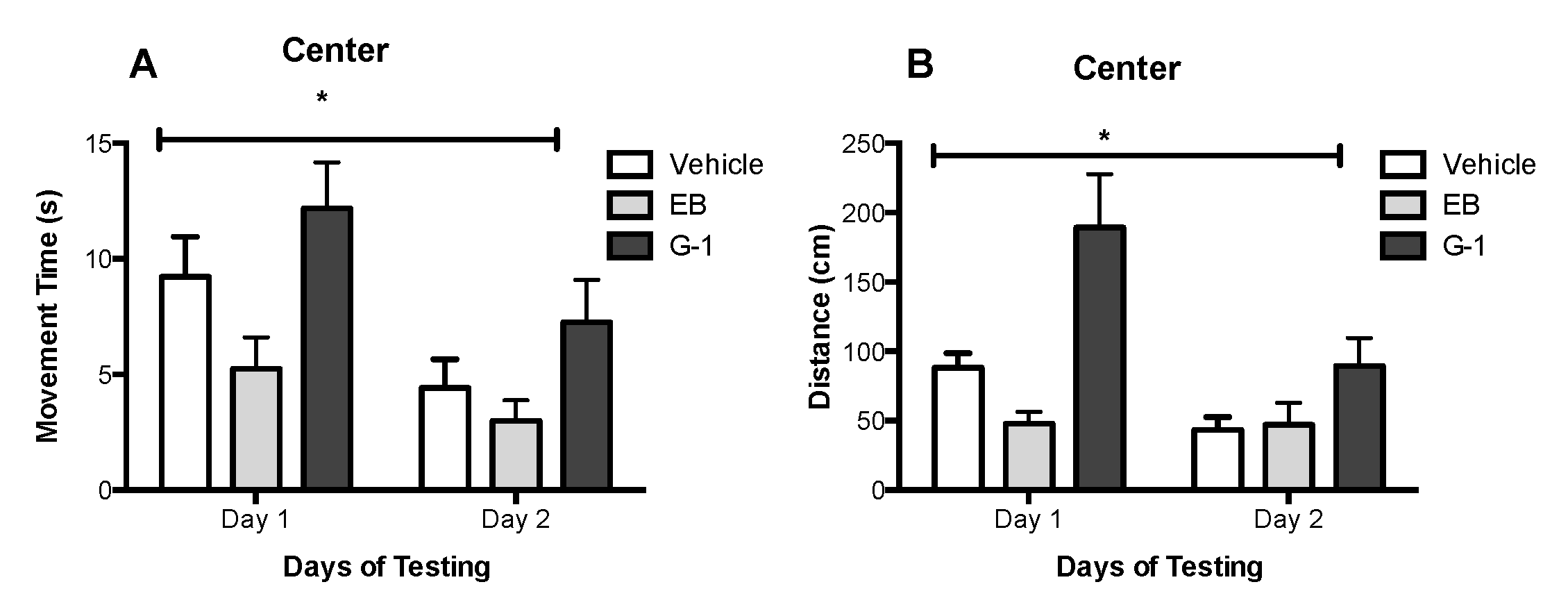

Supplement: Figure S1 — Chronic EB or G-1 treatment does not affect habituation to the open field over consecutive days. Animals administered EB or G-1 chronically in silastic implants were tested on two consecutive days to investigate habituation to the open field arena. Both movement time (A) and distance (cm) decreased during Day 2 compared to Day 1, irrespective of hormone treatment (*P < 0.05 cf days). Data represent mean ± SEM. [file brb30004-0051-sd1.tiff]
